# Oncocytic Variant of Medullary Thyroid Carcinoma - A Case Report

**DOI:** 10.30699/IJP.14.1.83

**Published:** 2018-12-27

**Authors:** Srilatha Parampalli Srinivas, Jayitri Das, Vidya Monappa

**Affiliations:** 1 *MBBS, MD Pathology, Associate Professor, Dept. of Pathology, Kastura Medical College, Manipal University, Manipal, Karnataka, India*; 2 *MBBS, Post-Graduate, Dept. of Pathology, Kasturba Medical College, Manipal University, Manipal, Karnataka , India*; 3 *MBBS, MD Pathology, DNB Pathology, Associate Professor, Department of Pathology, Kasturba Medical College, Manipal University, Manipal, Karnataka, India*

**Keywords:** Medullary Thyroid Carcinoma, Thyroid neoplasm, Fine-Needle Aspiration

## Abstract

Medullary thyroid carcinoma (MTC) is a rare tumor arising from parafollicular C-cells. The oncocytic variant of MTC is an extremely rare diagnosis, with less than 20 cases reported. Here we present the case of a 36-year-old male patient with complaints of neck swelling and dysphagia. On fine needle aspiration cytology (FNAC), a Hürthle cell neoplasm was suggested. Finally with histopathology and immunohistochemistry (IHC), a diagnosis of MTC oncocytic variant was established. This tumor can be easily misdiagnosed for any thyroid Hürthle cell lesions. An accurate diagnosis is important because MTC has different treatment protocols, and its oncocytic variant is expected to be associated with poorer patient survival. Thus, the oncocytic variant of MTC is a difficult diagnosis on FNAC. Histopathology and rel- evant IHC markers are necessary for a correct diagnosis.

## Introduction

Medullary thyroid carcinoma (MTC) is a rare tumor arising from parafollicular C-cells (1). It accounts for only 5-10% of all thyroid cancers. An oncocytic variant of medullary thyroid carcinoma is an extremely rare variant, and was first described by Harach in 1988 (2). Only less than 20 cases have been reported in his paper relevant literature.

Oncocytic change is seen in both benign and malignant diseases of the thyroid (3). However it is an uncommon finding in medullary thyroid carcinoma. This variant can be easily misdiagnosed for any other Hürthle cell lesions of the thyroid. Since medullary thyroid carcinoma has a different treatment protocol and its oncocytic variant is ex- pected to be associated with poorer survival, it is essential to make a correct diagnosis.

Literature states that the oncocytic variant of medullary carcinoma occurs in older patients and is more common in females. However, in our case the patient was a young male. It has also been stated that diagnosing oncocytic medullary thyroid carcinoma by fine needle aspiration cytology (FNAC) is extremely difficult, and this was found to be true in our report.

## Case Report

A 36-year-old male patient presented with complaints of neck swelling and dysphagia. Thyroid function tests revealed low T3 and T4 levels and mildly high Thyroid Stimulating Hormone (TSH) levels. Serum calcium was normal.

**Figure 1:**
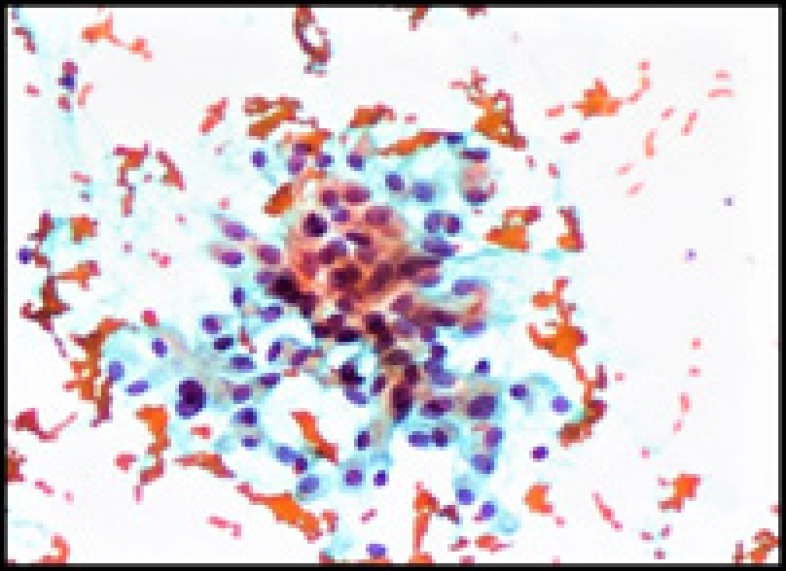
FNAC showing cluster of oncocytic cells (PAP stain, 100X)

FNAC findings suggested a Hürthle cell neoplasm with findings of oncocytic cells with eosinophilic cytoplasm, enlarged pleomorphic nuclei with nuclear crowding, overlapping and prominent nucleoli (Figure 1).

**Figure 2:**
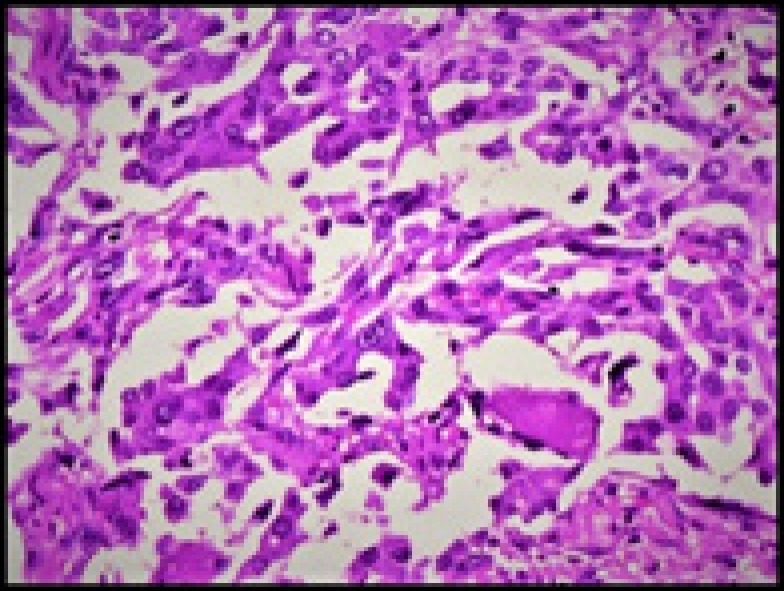
Nests of oncocytic tumor cells with amyloid in stroma (H & E, 100X)

**Figure 3:**
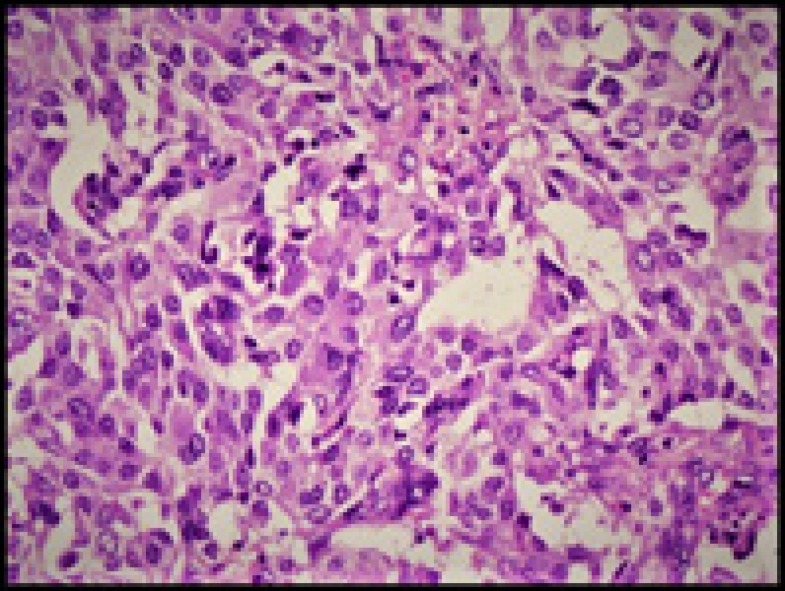
Polygonal oncocytic tumor cells (H & E, 200X)

**Figure 4:**
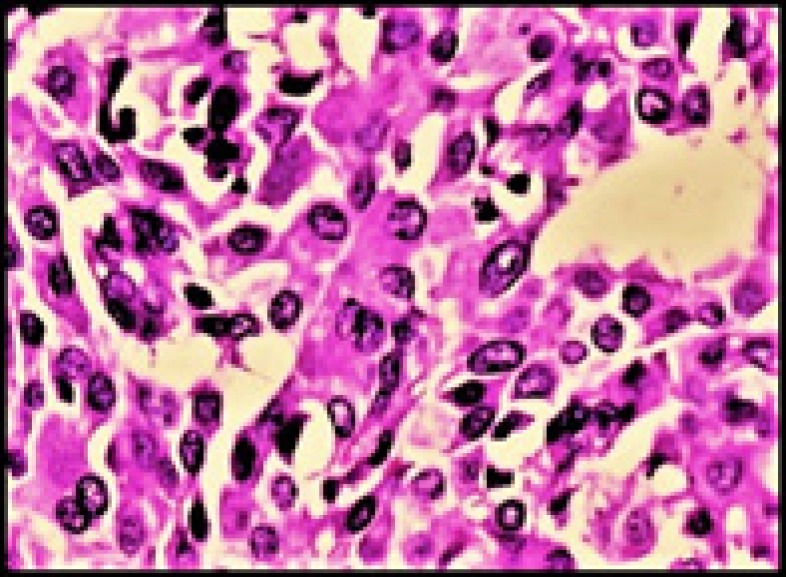
Oncocytic tumor cells with nuclei showing stippled chromatin (H & E, 400X)

Histopathology showed a malignant tumor comprised of nests of polygonal cells with abundant eosinophilic cyto- plasm, moderately pleomorphic nuclei, and stippled chromatin surrounded by spindly fibrous stroma with amyloid deposits (Figures 2- 4).

**Figure 5:**
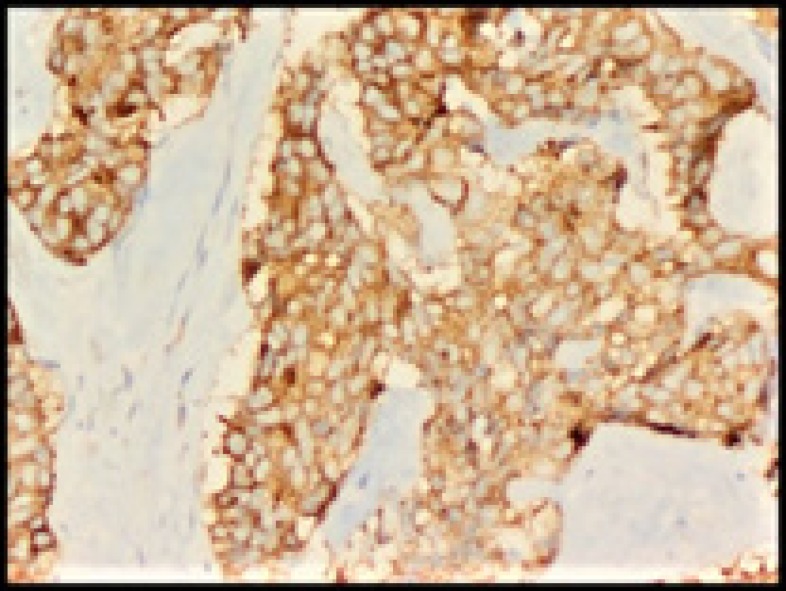
Synaptophysin-positive (100X)

**Figure 6:**
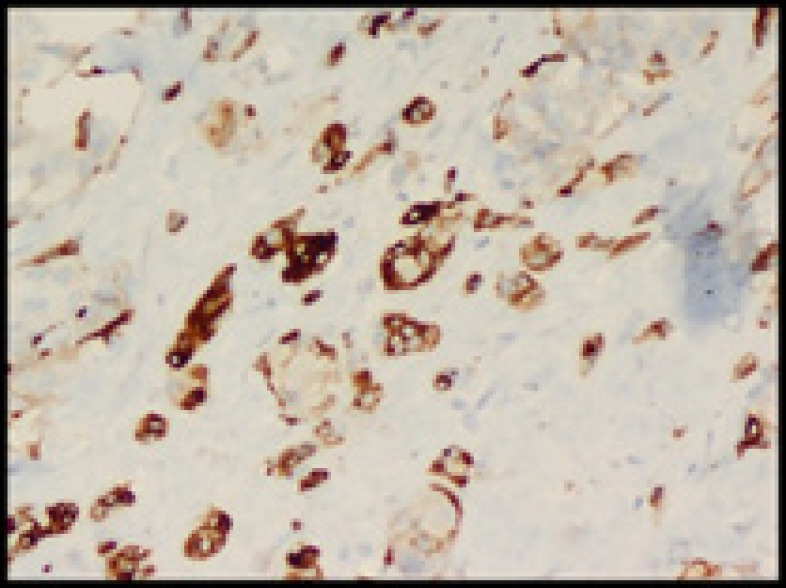
Chromogranin-positive (100X)

**Figure 7:**
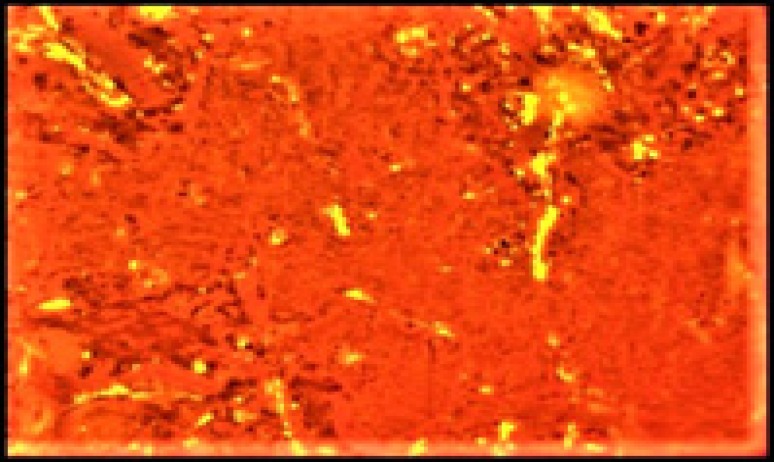
Congo-red positive (birefringence under polarizing light)

The tumor cells were positive for synaptophysin (Figure 5) and chromogranin (Figure 6), and amyloid was congo- red positive (Figure 7).

Based on histopathology and immunohistochemistry (IHC) reports, a diagnosis of the oncocytic variant of medul- lary thyroid carcinoma was given.

## Discussion

Medullary carcinoma is the third most common of all thyroid cancers (4, 5). It occurs in four clinical settings – 80% being sporadic, with the rest being part of MEN 2A, MEN 2B and familial medullary carcinoma thyroid. Inherited cases occur more commonly in younger patients than sporadic cases. Inherited MTC is autosomal dominant in nature, but some result from RET oncogene mutation as well. The latter are multifocal lesions while the sporadic cases, which also show RET oncogene mutation are single lesions.

MTC can present with a varied range of histologic patterns that were documented by Hazard in 1959. The described histomorphological patterns are classical spindle cell, papillary, pseudopapillary, follicular, clear cell, oncocytic, small cell, giant cell, paraganglioma-like, angiosarcoma-like, melanin-producing, squamous cell and amphicrine (6). Our case documents an extremely rare oncocytic variant of MTC.

The term “oncocytic MTC” was first coined by Harach and Bergholm in 1988 (2, 3). Subsequently, Domin- guez-Malagon (7) et al. in 1989 suggested that 60–70 % of the tumor cells should be oncocytic in nature to diagnose this variant.

Oncocytic cells have certain characteristic features on histology. These cells are large, polygonal, have abun- dant granular eosinophilic cytoplasm due to excessive mitochondria, and eccentrically located round to oval nuclei with prominent nucleoli. This is in addition to other regular features of MTC such as stippled chromatin and amyloid stroma. It is noted that other neuroendocrine tumors can also show oncocytic changes. Therefore, oncocytic change in medullary thyroid carcinoma is not an unexpected phenomenon, although it is rare (8-10).

Oncocytic change in the thyroid is more often associated with the follicular cells in both benign and malignant conditions. There can be a misdiagnosis of oncocytic MTC with any of these more prevalent conditions. Dif- ferential diagnoses of oncocytic MTC include Hürthle cell neoplasms, the oncocytic variant of papillary thy- roid carcinoma, oncocytic parathyroid neoplasms, oncocytic variant of poorly differentiated thyroid carcinoma, mixed follicular-medullary carcinoma with oncocytic cells, plasmacytoma, granular cell tumor and metastasis from tumors showing oncocytic cells, including paraganglioma, hepatocellular carcinoma, neuroendocrine car- cinoma, renal cell carcinoma, and malignant melanoma (3).

An important clue for diagnosing this tumor is the neuroendocrine type of stippled chromatin, as stated before. FNAC is not always conclusive, as in our case it was diagnosed as a Hürthle cell neoplasm on cytology.

On IHC calcitonin, carcinoembryonic antigen (CEA) and neuroendocrine markers like synaptophysin and chromogranin would be positive, while thyroglobulin would be negative. Amyloid stroma is demonstrated by congo-red positivity, as was shown in our case. Biochemical studies may show low serum calcium levels.

## Conclusion

The oncocytic variant of medullary thyroid carcinoma is a difficult diagnosis on FNAC. Histopathology and relevant IHC markers are necessary for a conclusive diagnosis. Since oncocytic change in MTC is associated with poorer prognosis, it is essential to make a right diagnosis for appropriate treatment.
